# Chemical Modulation of Mitochondria–Endoplasmic Reticulum Contact Sites

**DOI:** 10.3390/cells9071637

**Published:** 2020-07-07

**Authors:** Ana Paula Magalhães Rebelo, Federica Dal Bello, Tomas Knedlik, Natasha Kaar, Fabio Volpin, Sang Hun Shin, Marta Giacomello

**Affiliations:** 1 Department of Biology, University of Padua, Via U. Bassi 58/B, 35121 Padua, Italy; anapaula.magalhaesrebelo@phd.unipd.it (A.P.M.R.); federica.dalbello@gmail.com (F.D.B.); tomknedlik@gmail.com (T.K.); natasha.kaar@studenti.unipd.it (N.K.); fabio.volpin.1@studenti.unipd.it (F.V.); 2declan@gmail.com (S.H.S.); 2 Department of Biomedical Sciences, University of Padua, Via U. Bassi 58/B, 35121 Padua, Italy

**Keywords:** mitochondria–endoplasmic reticulum contact sites, mitochondria-associated membranes, pharmacology, drug targets, synthetic and biological compounds, neurodegeneration, diabetes, cancer

## Abstract

Contact sites between mitochondria and endoplasmic reticulum (ER) are points in which the two organelles are in close proximity. Due to their structural and functional complexity, their exploitation as pharmacological targets has never been considered so far. Notwithstanding, the number of compounds described to target proteins residing at these interfaces either directly or indirectly is rising. Here we provide original insight into mitochondria–ER contact sites (MERCs), with a comprehensive overview of the current MERCs pharmacology. Importantly, we discuss the considerable potential of MERCs to become a druggable target for the development of novel therapeutic strategies.

## 1. Introduction

Mitochondria–endoplasmic reticulum contact sites (MERCs) have been so far the primary actor in the scene of organelle contact sites. The latter are intracellular microenvironments “delimited” by the juxtaposition of two (or more) organelles, where signals from diverse transduction cascades converge, are integrated, and are sent to other subcellular structures. The reason why MERCs are so heavily studied is historical. Since their discovery, mitochondria and endoplasmic reticulum (ER) have been immediately recognized as fundamental for cell physiology—the former, as “energy factories”, the latter, as the site for protein folding first and main store of intracellular Ca^2+^. Additional contact sites are beginning to be studied, and this will certainly continue in the future, due to emerging techniques and increased knowledge about their role and the role of other organelles in cell pathophysiology [1].

A full description of MERCs composition, structure, and functions is beyond the scope of this review, and it has been covered by previously published articles, so we refer the reader to the appropriate literature [1,2,3].

However, we need to point out a few characteristics of MERCs here.

First, they are highly dynamic interfaces, undergoing changes upon stress or metabolic cell requests. This structural plasticity is highly connected to their functionality, that is, changes in MERCs organization guarantee the fine tuning of the pathways that they modulate, impacting not only cell physiology, but also that of the whole organism. For example, the MERCs resident protein mitofusin-2 (MFN2) is fundamental in shaping the job of pro-opiomelanocortin (POMC) neurons and its ablation leads to leptin resistance and whole-body energy imbalance [4]. This effect is specifically linked to the tethering function of MFN2 at MERCs, although MFN2 possesses other subcellular tasks such as participation in the mitochondrial fusion process. Hence, identification of a compound able to modulate MFN2-related MERCs functions in POMC neurons may represent an interesting approach to treat obesity [4].

Second, MERCs are highly tissue specific. According to previously published mass spectrometry analyses, proteins enriched at MERCs vary markedly, depending on the organ from which they had been extracted (Table S1, Supplementary Materials). This tissue specificity is further highlighted by the fact that mutated forms of ubiquitously expressed MERCs proteins result in organ-specific pathologies. For example, the brain is the primary region altered in Alzheimer’s and Parkinson’s disease caused by mutated presenilin-1/2 and parkin, respectively, which have been shown to reside at MERCs and impair Ca^2+^ exchange between the two organelles [3,5].

The third peculiarity that we would like to highlight is the ensemble of structural properties of MERCs: (i) the length of the parallel juxtaposition between the outer mitochondrial membrane and the ER surface; (ii) the distance separating the (surfaces of the) two organelles; (iii) the amount (i.e., the number) of contact sites occurring in a cell at a well-defined condition; and (iv) the persistence of these contact sites (e.g., how long they “last”) [2]. As happens in many biological systems, structural parameters have an impact on MERCs functions; we have recently proposed that MERCs function could be dual, that is, “vertical” or “horizontal” [6]. In the first case, we refer to processes demanding the intimate physical proximity of the two organelles, such as phosphatidylcholine synthesis, which requires the precursors to be transferred between the two organelles [7]. The second term refers to the activity of enzymes located at MERCs interfaces that account for the quantity of molecules here produced or processed, as in the case of cholesteryl esters [7]. Unfortunately, this “structure and function relationship” has not yet been clarified, but future studies are guaranteed.

Because of these structural features and their pleiotropic nature, MERCs (and more in general all membrane contact sites) appear as a highly complex system and a difficult target for drug discovery. However, we believe they should be taken into account in the new branch of pharmacological research known as structural systems pharmacology, which considers not only the specific properties of the drug targets, but also their environment [8,9].

Although some drugs target MERCs resident proteins, either by direct binding or indirectly through the modulation of their expression levels, the pharmacological profile of MERCs has been neglected. In this review, we first summarize the main disorders linked with MERCs defects and then provide a compendium of the compounds described so far that are able to modulate MERCs function or structure. We also speculate on the possibility that chemical modulators of organelle contact sites are the next frontier in pharmacology—in this context, not a single molecule but a whole set of juxtaposing membranes (composed of specific subsets of lipids and proteins) will be exploited as a novel druggable target.

## 2. MERCs and Neurodegenerative Disorders

Most of the MERCs-linked disorders include neurodegenerative symptoms, either at the central nervous (CNS) or peripheral nervous system (PNS). Despite the finding that mutated forms of MERCs resident proteins are the genetic determinants of some disorders, the respective cause-and-effect relationship is still obscure: do MERCs changes actively participate to disease onset, or are they a consequence of the pathological condition? In some diseases, the causative role of MERCs changes is supported by several lines of evidence.

One of the most interesting examples is that of amyotrophic lateral sclerosis (ALS). Indeed, among genetic causes of ALS are mutations of the valosin-containing protein (VCP), vesicle-associated membrane protein-associated protein B/C (VAPB), protein tyrosine phosphatase interacting protein 51 (PTPIP51), and the TAR DNA-binding protein 43 (TDP-43) [10,11]. These proteins have two common traits: first, they are involved in the control of autophagy; second, they all reside at MERCs [2,5,11]. As MERCs have been recently identified as a site of autophagosome formation, it is likely that MERCs-mediated autophagy plays a key role in the pathogenesis of ALS [1,11]. Other MERCs functions appear disrupted by mutated forms of VAPB/PTPIP51/TDP-43; for example, overexpression of TDP-43 is sufficient both to lower ER–mitochondria Ca^2+^ exchange and to mimic (in rodents) the disease phenotype caused by a mutation in the 3’ untranslated region of the *TDP-43* gene that enhances its expression level [10,11]. Another MERCs protein known as sigma-1 receptor, whose mutations are linked to familial ALS cases, has also been reported to decrease mitochondria–ER crosstalk [12], reinforcing the causative role of MERCs in ALS. 

Another example is that of Alzheimer’s disease (AD). Since its discovery, several hypotheses have been proposed to explain its underlying molecular mechanisms. First, we note the beta-amyloid (Aβ) cascade hypothesis, which suggests that neuronal death is caused by the accumulation of extracellular plaques of Aβ, the cleavage product of the amyloid precursor protein (APP) in the brain [13]. Second, there is the Ca^2+^ hypothesis, where changes in intracellular Ca^2+^ homeostasis cause all the subcellular defects (altered lipid and Ca^2+^ signaling, mitochondrial dysfunctions, increased susceptibility to cell death) described in both patient- and mice model-derived cells [14]. However, the evidence that lipid metabolism/synthesis was also imbalanced in AD patients called for alterations at the crossroad among all these pathways. MERCs, also known as mitochondria-associated ER membranes (MAMs, if isolated through biochemical fractionation [2]) appeared to be the ideal candidate, leading to the formulation of the MAM hypothesis [15]. This theory is corroborated by the fact that the core subunits of the APP cleavage complex, the presenilin proteins, are enriched at MAMs where Aβcan also be produced.

Another neurodegenerative disorder associated with MERCs defects is Parkinson’s disease (PD). PD is characterized by the death of dopaminergic neurons and by the presence of cytosolic aggregates of alpha-synuclein [16]. The latter has been retrieved at MAMs, where its PD-associated mutants impair both MERCs structure and Ca^2+^ transfer between the two organelles [17]. Furthermore, most genes responsible for inherited PD cases either reside at MERCs or modulate them, e.g., parkin, whose overexpression increases interaction between the two organelles [18,19].

Defective organelle function also affects peripheral nerves, leading to motor and sensory peripheral neuropathies (PNs [20]). The family of PNs includes a heterogeneous group of diseases characterized by loss of sensitivity and autonomic nervous system dysfunctions. Some forms of inherited PNs, known as Charcot–Marie–Tooth disease (CMT), are also linked to defective MERCs, no matter whether their phenotype is mainly demyelination or axonal degeneration of motor neurons [6,20,21]. Accordingly, the axonal CMT type 2a is caused by MFN2 mutations impairing its mitochondria–ER tethering activity and, consequently, the cholesteryl ester/Ca^2+^ homeostasis [6,22,23]. Ganglioside-induced differentiation-associated protein 1 (GDAP1) and diacylglycerol O-acyltransferase 2 (DGAT2), responsible for demyelinating CMT4A and axonal CMT2 disorders, respectively, have also been retrieved at MAMs [21,24]. Interestingly, changes in the expression levels of GDAP1 and DGAT2 compromise MERCs architecture and function, which further sustains a possible link between the onset of PN and dysfunctional organelle crosstalk [21,24,25].

Another example of a MAM-linked disorder is hereditary spastic paraplegia (HSP). Its main genetic cause are mutated forms of ER-shaping proteins, such as receptor expression-enhancing protein 1 (REEP1), atlastin, spastin, and strumpellin [26]. Mutants of these proteins have been reported to alter organelle contact sites, either involving mitochondria or not. As an example, REEP1 resides at MERCs, where it positively controls organelle interaction. Its HSP-linked mutants instead fail to do so and impair neurite growth and axon function [27]. Atlastin, REEP1, and strumpellin mutants also change the interaction between ER and endosomes, which is necessary for endosomal tubule fission [28]. Whether the endosome–ER contact alterations are a consequence or a cause of MERCs defects in HSP remain to be established.

Even rarer disorders are linked to MERCs defects; for example, the MEGDEL syndrome caused by mutated serine active site-containing protein 1 (SERAC1 [29]). Features of this disorder include methylglutaconic aciduria (MEG), deafness (D), encephalopathy (E), and Leigh-like disease (L). At MERCs, SERAC1 controls the remodeling of the cardiolipin precursor phosphatidylglycerol; its mutant forms thus impair mitochondrial and in turn cell physiology [29,30].

As underlined by the former evidence (for more details, refer to [1,2,31]), many MERCs-associated conditions belong to the spectrum of neurodegenerative diseases. This is likely due to the peculiar structure of neural cells, including neurons, astrocytes, and oligodendrocytes. For example, the shape of neurons “determines” their function, as axons and dendrites convey electrochemical signals [32,33]. Thus, processes that influence the MERCs will in turn modify neuronal cell physiology and functionality, impacting the overall biology of the CNS. While this rule holds true for all cell types, it is likely to fit even more tightly to neural cells, because each CNS cell is unique in its shape, position, and ability to transmit signals to other brain cells or compartments. Any subcellular alteration (including MERCs defects) ending up in disrupting cell extensions (as axons or dendrites) is thus likely to impact the overall CNS cell connectivity and signaling. Similarly, the “wiring economy” of neurons [34,35] is also to be affected, proportionally to the extent of the MERCs damage.

## 3. MERCs and Metabolic Disorders

Besides MERCs role in CNS disorders, much evidence underlines their contribution also to metabolic syndromes. Two key functions of these interfaces must be taken into account in this context.

First, we note their contribution to lipid homeostasis.

For example, the formation of phosphatidylcholine (PC) starts with the synthesis of phosphatidylserine (PS) in the ER, which is then transferred into mitochondria where it is converted into phosphatidylethanolamine (PE). The latter is then transported back to the ER where it is converted into PC. It has been shown that abnormal PE production impairs autophagy, as covalent attachment of PE to the autophagy protein Atg8 is pivotal for the formation of autophagosomes [36,37,38,39]. Altered phospholipid synthesis, due to the defective PS exchange at MERCs, has been shown as the key mechanism underlying a widespread human disorder, non-alcoholic steatohepatitis [40]. Even steroidogenesis, the process through which steroid hormones are produced from cholesterol, is controlled by MERCs. This is fostered by cholesterol import into mitochondria, mediated by the interaction of the two MERCs resident proteins, namely voltage-dependent anion-selective channel protein 2 (VDAC2) and steroidogenic acute regulatory protein (StAR) [41]. Additionally, lipoid congenital adrenal hyperplasia, an endocrine lethal disorder, is caused by the defective import of cholesterol into mitochondria caused by mutated forms of StAR [42].

The second MERCs function is that of being the site at which ER and mitochondria exchange Ca^2+^ [2]. Indeed, the activity of three mitochondrial dehydrogenases depends on the levels of Ca^2+^ in the mitochondrial matrix. Increased Ca^2+^ concentration fosters the citric acid cycle, hence increasing NADH levels and ATP production [43]. Notably, changes in the matrix Ca^2+^ concentration indirectly control fatty acid β-oxidation; by regulating the levels of acetyl-CoA, the key β-oxidation enzyme 3-ketoacyl-CoA thiolase is inhibited [44].

MERCs-mediated Ca^2+^ transfer is therefore fundamental for the cell to switch from glucose metabolism to fatty acid oxidation, a possibility which is often referred to as “metabolic flexibility”. As such, altered Ca^2+^ transfer would result in metabolic inflexibility, a condition typical for metabolic disorders such as obesity, insulin resistance (IR), and diabetes (type 2 especially) [45]. Several pieces of experimental evidence further corroborate the finding that defective MERCs (more specifically, impairment of their Ca^2+^- and phospholipid-related tasks) underlie these pathological conditions [43].

For instance, a mouse knockout model for cyclophilin D, a mitochondrial protein that likely interacts with the multiprotein complex responsible for Ca^2+^ transfer (especially with VDAC), is characterized by a lower number of contacts between the two organelles and by hepatic IR. Interestingly, restoring the MERCs structure has been enough to rescue IR [46]. In line with this, genetic manipulation of MERCs by expression of fetal and adult testis-expressed 1 (FATE-1) dampened insulin response in rat hepatocytes and mouse livers [47].

Mice harboring liver-specific mutation of inositol 1,4,5-triphosphate receptor 1 (IP3R1), the Ca^2+^ releasing unit of the ER, display hyperglycemia and higher susceptibility to dietary-induced diabetes [48]. Not only liver but also skeletal muscles of these animal models are characterized by a lower number of organelle interactions that likely underlie the subsequent mitochondria dysfunctions. Similar evidence was retrieved in myotubes isolated from obese patients and from individuals affected by type 2 diabetes [49,50,51].

In contrast, hepatocytes from different models of obese mice (leptin-deficient and diet-induced) are characterized by enhanced mitochondria–ER proximity [52] and altered mitochondrial dynamics, which could be counteracted (at least in cardiomyocytes) by melatonin administration [53].

Interestingly, IR could be a consequence not only of lower interaction between the organelles, but also of its increase. Indeed, it has been recently shown that livers from obese mice are characterized by overexpression of phosphofurin acidic cluster sorting protein (PACS2) and IP3R and by enhanced Ca^2+^-related MERCs function, and that insulin sensitivity can be ameliorated upon downregulation of these two proteins [52,54]. Although apparently contradictory, these results could be explained by the use of different experimental models (e.g., likely with different genetic background, potentially also impacting MERCs [55]) and by the fact that the analysis was performed in tissues with different metabolic requirements. In any case, these lines of evidence highlight the fact that interaction between the ER and mitochondria contributes to insulin and/or glucose signaling in liver and insulin-sensitive peripheral tissues. MERCs hyper- or hypo-association leads to the development or enhanced susceptibility to metabolic disorders [51]. Therefore, MERCs could be considered as a new intracellular target to handle insulin action and secretion as well as glucose dyshomeostasis in the context of metabolic diseases.

## 4. MERCs and Cancer

The study of MERCs involvement in the growth and metastatization of different types of cancer is an exponentially growing field. Here, we briefly summarize a few concepts of cancer research, being in our perspective one of the pathological scenarios where MERCs-targeting compounds could provide a big “therapeutic” step forward (in-depth description of cancer biology is beyond the scope of this review; therefore, we refer the reader to specific works such as [56,57]).

Oncogenes and tumor suppressors can benefit from the MERCs functions, as this platform can promote metabolic reprograming, restriction or hyperactivation of Ca^2+^-dependent signaling, antioxidant response, and apoptosis (reviewed in [58,59]). This is supported by the findings that the products of oncogenes or tumor suppressors have been found at the mitochondria–ER interfaces [59,60]. A well-established example is the promyelocytic leukemia protein (PML), encoded by a tumor-suppressor gene implicated in leukemia. At MERCs, PML controls the phosphorylation state of IP3R and hence Ca^2+^ transfer between ER and mitochondria. Under normal conditions, PML modulates the activity of the serine/threonine kinase Akt by recruiting the protein phosphatase 2a (PP2a); Akt inhibits IP3R-mediated Ca^2+^ release by phosphorylating it, whereas PP2a counteracts this event [61]. Therefore, mutations affecting the PML gene (and resulting in lower expression of PML) hamper the recruitment of PP2a and lead to hyper-phosphorylation of IP3R and reduction of ER Ca^2+^ release as well as to susceptibility to pro-apoptotic stimuli [62,63]. The role of PML at MERCs does not concern only Ca^2+^ homeostasis, as PML loss has also been linked to hyperactivation of autophagy [61]. This could also contribute to its carcinogenic activity, as sustained autophagy has been described in many types of cancer as a way to fulfill the energetic demands associated with high cellular proliferation rates and survival within restrict environments.

The tumor suppressor p53 was also found at MERCs, where it regulates the activity of the sarco-endoplasmic reticulum Ca^2+^ ATPase (SERCA), the pump responsible for Ca^2+^ re-uptake into the ER upon its release into the cytosol [64]. Other examples of MERCs resident Ca^2+^ modulators described with pro- and anti-oncogenic actions include phosphatase and tensin homolog (PTEN), breast cancer type 1 (BRCA1), and B-cell lymphoma 2 (BCL-2) (reviewed in [58]).

One of the pro-survival oncogenic adaptations is the increased resistance to apoptosis; in particular, changes in the localization or expression of proteins that modulate Ca^2+^ efflux from ER to mitochondria can interfere with the opening of the mitochondrial permeability transition pore (mPTP) [65], which in turn causes mitochondrial depolarization and activation of cell death programs.

The interplay between MERCs and cancer is nowadays highly studied. Understanding the implication of MERCs in the initiation or progression of tumors and metastasis is highlighting new molecular targets in chemotherapeutic drug development.

## 5. Mechanisms for Chemical Modulation of MERCs

**Figure 1:**
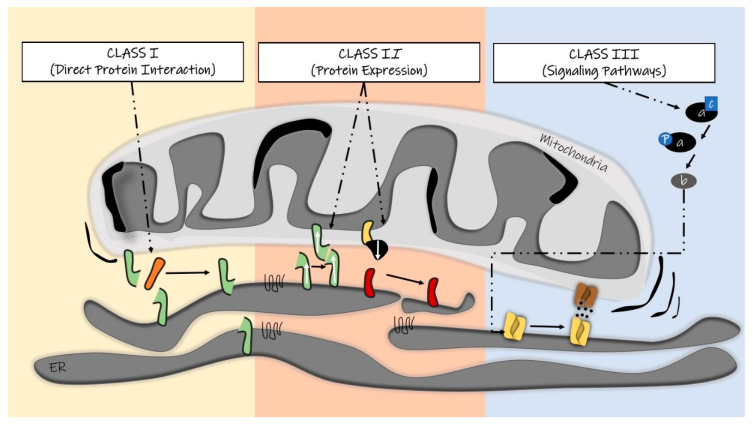
Schematic of the three classes of compounds that could modulate mitochondria–endoplasmic reticulum contact sites (MERCs) structure and function.

The classical pharmacological approach infers the presence of a target (commonly proteins, sugars, or lipids) for the design and development of a specific drug. Compounds are designed to (pro)fit into a protein scaffold, thus either hampering its interaction with other partners or cofactors or blocking its (enzymatic) activity. It is quite obvious, however, that MERCs pharmacological modulation is more complex than this. We tried to rationalize the possible MERCs modulators by clustering them into three classes: (i) compounds characterized by direct interaction with proteins located at MERCs, especially those responsible for organelle tethering (ii) molecules inducing changes in the expression levels of MERCs resident proteins; and (iii) compounds targeting signal transduction cascades that ultimately lead to changes in MERCs structure or function (Figure 1).

In the following sections, we sum up the current literature regarding each of these classes.

## 6. MERCs Modulator Class I: Targeting MERCs Structural Components

As stated above, due to the outgrowing interest in organelle contact sites, we have now access to an extensive list of molecules known to control or compose these heterotypical membrane proximities. One of the most explored groups of molecules are those able to connect two organelles, namely the tethers. They are defined as resident or transitory elements that physically connect the surfaces of the two organelles, by means of protein–protein or protein–lipid interactions [66]. Tethers are also assumed to possess specific molecular functions, e.g., participating in the transfer of ions and lipids [67]. Therefore, they could be considered as a second signaling messenger, which shapes the cell physiology by adjusting the distance, length, number, and localization of MERCs [68]. Our increasing knowledge about the identity of these tethering structures, as well as about their structural and functional roles, makes them promising biological targets for chemical modulators.

A well-described MERCs tethering complex is composed of the ER protein vesicle-associated membrane protein-associated protein B (VAPB) and the mitochondrial protein tyrosine phosphatase-interacting protein 51 (PTPIP51) [69]. Modulation of VAPB or PTPIP51 directly impacts the ER–mitochondria contacts distance, that is, their overexpression or loss results in MERCs tightening or loosening, respectively [69]. In terms of cell functionality, the VAPB–PTPIP51 complex has been described to be involved in Ca^2+^ exchange between the two organelles with implications for basal and chemical-induced autophagy progression [70]. These findings reinforced the idea that MERCs can shape autophagy, a cellular process of great interest in terms of chemical modulation [71,72]. The recent discovery of a small molecule named LDC-3/Dynarrestin that directly targets PTPIP51 is in this review’s interest [73]. First, this aminothiazole was characterized by its antagonistic actions in cytoplasmic dynein with consequent disturbance of the Hedgehog signaling [73]. Later on, LDC-3 was found to have high binding affinity to PTPIP51 during a small molecule high-throughput screen, with implications for PTPIP51 downstream signaling [74]. Although LDC-3 was shown to increase the interaction between PTPIP51 and VAPB, its effects on MAM biology have not been explored. Nevertheless, it is possible that LDC-3 itself or its analogs can be exploited as a MERCs-targeting molecule of relevance to neurodegeneration and cancer-related pharmacological research.

Another complex, composed of the inositol 1,4,5 triphosphate receptor (IP3R)/glucose-regulated protein 75 (GRP75)/voltage-dependent anion channel (VDAC), drives Ca^2+^ exchange at MERCs [75]. IP3R is a calcium channel located on the ER membrane that controls Ca^2+^ efflux from the ER into the cytosol. At points of high proximity with mitochondria, the amount of Ca^2+^ released as well as its efflux and reuptake rates shape the formation of microdomains of high Ca^2+^ concentration on the surface of mitochondria. The outer mitochondrial membrane protein VDAC, coupled to the inner mitochondrial membrane Ca^2+^ uniporter (MCU), drives Ca^2+^ entry [2,41,66]. IP3R is bound to VDAC through GRP75. Molecules able to change their interaction could therefore be exploited as therapeutics to correct eventual MERCs-dependent Ca^2+^ defects [62].

Of note, many compounds target VDAC1 and modulate its activity (reviewed in [76,77,78]). For example, König’s polyanion (KPa) can induce VDAC1 closure in vitro, although in live cells it works either as a pro- or anti-apoptotic drug, depending on the cell type and experimental condition [79]. The commonly used anti-inflammatory compound aspirin also targets VDAC, promoting apoptosis through mitochondria depolarization, suggesting its potential use for cancer treatment [80]. Similarly, the class of compounds known as “avicins” leads to VDAC inhibition, cytochrome c release, and cell death [81,82].

Other chemicals impinge on VDAC activity, although not through its direct inhibition, but by blocking its interaction with partner molecules, e.g., hexokinase (HK) and the adenine nucleotide transporter (ANT). Such molecules include antimycotic drug clotrimazole [83], pyruvate analogue 3-bromopyruvate [84], and the plant stress hormone methyl jasmonate, which all induce detachment of HK from VDAC and stimulate apoptosis [85]. In addition, a selective peptide was shown to dislocate HK from MAMs in colon and breast cancer cells, and consequently to induce mitochondria Ca^2+^ overload [86]. With respect to ANT, its association with VDAC is disrupted by arsenites, ionidamine, and steroid analogs [87].

Another example of direct pharmacological modulation concerns mitofusin-2 (MFN2). It is characterized by different conformational states; phosphorylation of a serine residue favors an “open” conformation, which enables MFN2 pro-fusion activity. Recently, small molecules or mini-peptides mimicking the peptide interface involved in this structural change have been identified as MFN2 agonists, promoting its fusogenic activity [88]. The specificity of these molecules is a premise for the treatment of MFN2-associated disorders (e.g., inherited peripheral neuropathy Charcot–Marie–Tooth disease type 2A (CMT2A) [89]) and for the possibility to control the different cell pathways in which MFN2 is involved. While this protein specificity is promising, a careful evaluation of the subcellular effects of these drugs is needed. Generally speaking, proteins participate in many processes, and this is also the case of MERCs resident proteins. As a consequence, different subcellular pathways are modulated at once, underlying potential side effects. Whether a compound is able to control directly the MERCs-associated function of its target needs to be carefully evaluated from two points of view—on the one hand, through in vitro assays to estimate the (enzymatic) activity of the target protein or its binding to the drug (e.g., with nuclear magnetic resonance spectroscopy) and, on the other hand, by measuring the effects of compounds on MERCs structure (electron and fluorescence microscopy approaches [90]) as well as function (measurement of Ca^2+^ and lipids transfer). These approaches will highlight potential side effects due to the presence of different protein variants—with different functions—or to different localization (as an example, MFN2 resides both at the ER and outer mitochondrial membrane). Therefore, once selective inhibitors are available, a “second selectivity step” to benefit only from MERCs resident variants would be needed. To our knowledge, this challenging task has not been addressed yet. A possible solution could be to generate compounds with dual specificity, e.g. targeting two MERCs resident proteins at once.

## 7. MERCs Modulator Class II: Transcriptional Modulators

The second type of MERCs modulators encompasses those affecting expression levels of MERCs resident proteins, likely by acting on transcription factors that promote the RNA synthesis of MERCs components. These compounds have obvious specificity issues, unless transcription factors play a selective role and are not generally used for many genes.

A wide range of plant-derived compounds have been described with implications for MFN2 expression, including the crude flavonoid extract from *Erigeron breviscapus* (named breviscapine) [91], the glucoside salidroside [92], the anti-inflammatory and anti-oxidant polyphenol resveratrol [93], and nicotine [94].

For example, treatment of some cancer cell lines with resveratrol, or with another polyphenol, piceatannol, has been shown to alter both structurally and functionally MERCs, i.e., a 36-h exposure of the cells to these compounds enhanced ER–mitochondria tethering and Ca^2+^ transfer between the two organelles, thereby inducing cell death [95]. Curiously, in some cell types resveratrol can trigger autophagy, in an IP3R-dependent manner, likely by passive leak. This in turn dampens agonist-induced Ca^2+^ release and alters physiological signaling pathways [96]. Besides modulating the expression of MERCs resident proteins, resveratrol has been reported to activate or repress several transcription factors such as AP-1, CREB, Egr-1, Elk-1, and Nrf2, in a cell-type specific manner [97], which could be at the basis of undesired side effects.

Another mixture of compounds with described effects on MERCs-associated proteins is breviscapine. It has been explored for its protective action in models of hepatic, neuronal, and cardiac ischemia reperfusion (I/R) [91,98,99]. Breviscapine upregulates MFN2 expression in hepatocytes during I/R, and in parallel exerts protective functions through a poorly explored mechanism. Indeed, MERCs dynamics in response to I/R are not yet clear, and could be part of a feedback loop initiated by other I/R signaling pathways [100]. Understanding the effect of breviscapine on MERCs, and how the targeting of these interfaces can have an impact on I/R injury, will provide valuable therapeutic insights.

Another transcriptional modulator of MERCs is the alkaloid berberine, already known for its broad effects on metabolism, particularly the induction of glycolysis and fatty acid β-oxidation [101,102,103]. Berberine injection in a mouse xenograft model resulted in decreased levels of MFN2 as well as reduced complex I activity and mitochondrial membrane potential [101,102,103].

Metformin, a well-described antidiabetic drug with multi-organ/tissue benefits in terms of glucose metabolism and production, was also able to revert the disruption of MERCs structure observed in high-fat and high-sucrose diet-induced insulin-resistant mice, mediated by lower expression of VDAC1 and PACS2 and higher levels of MFN2 [104,105]. Another compound, found in broccoli sprouts and named sulforaphane, has been proposed as an antidiabetic treatment for its ability to increase MAM protein content, diminish ER stress markers, and restore the VDAC1–IP3R1 interactions in the same murine models. Interestingly, cumulative pieces of evidence have enlightened the fact that mitochondria–ER interactions are the key player in the metabolism of hepatocytes. Moreover, chemicals able to modulate MERCs structure appear curative of insulin resistance (at least in experimental models). Altogether, other studies are therefore expected to come in the next years, to clarify MERCs contribution to the physiology of hepatocytes and to assess the effects of drugs for the treatment of liver disorders [49,106].

A vast number of chemotherapeutics is also known to indirectly modulate mitochondrial/ER functions at MERCs. These agents include anthracycline doxorubicin (brand name Adriamycin), cisplatin, trastuzumab (Herceptin), arsenic trioxide (Trisenox), and mitoxantrone (Novantrone).

Doxorubicin is a compound likely exerting MERCs-related effects, although this aspect has never been investigated in depth. This drug is widely applied for the treatment of leukemias, lymphomas, and solid tumors, although it is characterized by a dose-dependent toxicity in several organs (e.g., the heart, brain, liver, lung, and skeleton), cardiomyopathy being the most devastating side effect [107]. Of note, exposure of neonatal rat cardiomyocytes to doxorubicin caused oxidative stress and apoptosis, associated with lower MFN2 expression. Whether this is a tissue-specific effect (e.g., occurring only in heart) or it is a general mechanism of doxorubicin action remains to be defined [108]. Whether doxorubicin affects primarily MERCs or MERCs changes are a consequence of other subcellular targets is not clear. Indeed, doxorubicin can also decrease the expression of Bcl2, promoting oxidative stress and enhancing sensitivity to cell death stimuli in breast cancer cell lines [109].

Cisplatin is another anticancer drug with potential effects on MERCs homeostasis [110,111,112]. Indeed, its cytotoxic effect is either mediated or modulated by IP3R, in a complex feedback mechanism likely contributing to the development of cisplatin resistance [111]. Furthermore, a small molecule named ABT-737, able to inhibit Bcl-2, has been shown to restore cisplatin sensitivity by the upregulation of MFN2 and GRP75 levels [112,113,114].

Considering that doxorubicin and cisplatin are able to control different steps of the apoptotic cascade, their exact mechanism of action appears hard to establish. More specific (MERCs-related) experimental approaches will be necessary to dissect their complex subcellular effects. 

Accordingly, it must be noted that the transcriptional effects of the class II MERCs modulators could either directly change the levels of a certain MERCs resident protein or modulate a signaling pathway, which induces changes in the structure or function of these dynamic interfaces. 

The discovery of molecular MERCs modulators can transcend the screening of bioactive compounds by focusing on post-transcriptional regulatory molecules such as microRNAs (miRNAs). These non-coding small RNAs (20–23 nucleotides) entered their target mRNAs by recognizing sequences at its 3’-UTR, ultimately leading to inhibition of their translation or to degradation of the mRNA itself. This endogenous way of interfering with protein expression has been of great interest, since miRNAs are extremely specific and since their levels are altered under pathological conditions. miRNA mimetics or inhibitors appear to be a promising therapeutic application to modulate the expression levels of their targets [115]. As an example, several miRNAs have been shown to target the fission protein 1 (FIS1), which has been described to interact with B-cell receptor-associated protein 31 (Bap31) at MAMs following apoptotic stimuli [116]. Bap31 participates in the quality control system of the ER (e.g., specifically, in the ER-associated degradation, which ensures clearance of misfolded proteins). Its binding to FIS1 promotes Bap31 cleavage into a pro-apoptotic form (named p20) that supports Ca^2+^ transfer from the ER to mitochondria and mitochondrial fission followed by the cytochrome c release [117]. In this context, lowering FIS1 expression via miR-484 reduced mitochondrial fission and apoptosis under hypoxic conditions [118]. Likely miR-484-induced FIS1 downregulation lowers its interaction with Bap31; miR-484 mimetics could, therefore, represent an alternative strategy to shape the Ca^2+^-mediated pro-apoptotic function of MERCs.

## 8. MERCs Modulator Class III: Targeting MERCs through Modulation of Upstream Signaling

Several signaling pathways controlling MERCs architecture have been described. For example, it has been shown that the induction of ER stress enhances the interaction between the two organelles, as an adaptive mechanism [119]. The metabolic shift occurring in the liver upon feeding is accompanied by an increase in MERCs length [2,120], supporting the hypothesis that MERCs are fundamental in shaping mitochondrial metabolic function.

Normally, drugs exert two types of actions, namely therapeutic and side effect. Given the pleiotropic nature of MERCs and their ability to quickly adapt to intracellular pathways, it is obvious that drugs targeting key signaling players also influence their structure or function. Of course, this event might be either positive (e.g., reinforcing the curative effect) or negative (e.g., responsible for the generation of side effects), and might occur in a dose-dependent manner, according to the drug pharmacokinetics. In addition, as MERCs are modulable in a tissue-specific manner, the phenotypic outcome of a drug treatment might affect MERCs at different extents in different organs.

For example, lithium treatment, used in patients affected by bipolar disorder and likely exerting neuroprotective effects [121,122], changes the expression levels of several genes in astrocytes, including Krüppel-like factor 4 (KLF4) and PARK2 co-regulated (PARKRG) [123]. The protein products of these two genes are particularly interesting as they are involved in key processes, in which MERCs remodeling also takes part. In particular, the transcription factor KLF4 controls the expression of autophagic genes [124], while PARKRG can suppress cell death and induce autophagy [125,126]. Similarly, different doses of levodopa, used for the treatment of PD, induce homeostatic changes through transcriptional regulation of mitogen-activated protein kinase (MAPK) cascades:protein kinases (MAPK) cascades: these also account MERCs resident proteins as substrates to exert their functions related to cell differentiation and proliferation, mitosis, and cell survival/death [127,128].

Another molecule that can indirectly alter MERCs is quercetin, which has been shown to alter the activity of the AMP-activated protein kinase (AMPK) [129]. AMPK acts as a sensor of the energetic status of the cell, regulating processes or proteins that can promote ATP production [130]. Notably, AMPK affects the activity or expression of the MERCs resident protein thioredoxin-interacting protein (TXNIP) by direct phosphorylation or indirectly by blocking the DNA-binding region of its transcription factor carbohydrate response element-binding protein (CREB) [131,132]. Quercetin can therefore influence TXNIP function, especially the MERCs-related one, which is important for inflammatory response. Indeed, TXNIP accumulates at MERCs in response to oxidative stress and mediates production of interleukin (IL)-1β [129,133,134].

An interesting RNA sequencing screen highlighted the transcriptional changes occurring in primary cerebrocortical cultures from mice brain following treatment with clinically approved drugs [135]. The authors provide a compendium of the transcriptional changes induced by these conditions, which can be used to get insights on blood–brain barrier-penetrant therapeutics and provide a basis for drug repurposing. Besides using their database to identify drugs that can up-/downregulate MERCs resident proteins (e.g., according to their database, the compound rivastigmine, used to treat different types of dementia including AD, can upregulate MFN2 and downregulate sterol O-acyltransferase 1, (SOAT1) [135]), it will be possible to identify the different signaling pathways induced by these compounds. Although “influenced” by the cell type used and experimental conditions, datasets like this could shed light not only on additional therapeutic use but also on the mechanisms underlying the associated side effects.

As a final remark, it is worth noting that although the cell-type specificity mentioned above could be exploited to design tissue-specific therapeutics, normal cell toxicities could also occur. Here are two examples—first, the antitumor activity of a compound able to promote MERCs-mediated cell death of cancer cells could result in the reduced viability of normal cells; and second, a molecule able to decrease a disease-linked upregulated ER–mitochondria proximity could result in lower sensitivity to cell death and promote carcinogenesis.

## 9. Conclusions

Mitochondria–ER interactions appear to be the key players in a number of human pathologies. The possibility to exploit such dynamic interfaces as therapeutic target has not yet been explored, likely due to their complexity both in terms of structure and function. Nonetheless, compounds able to alter MERCs have been already described. In this review, we proposed their classification based on the type molecular targeting of the MERCs interface—first, direct interaction of small molecules of synthetic or biological origin with well-described MERCs resident proteins (in other words, disrupting or enhancing the interaction of tethering proteins with protein or lipid); second, compounds inducing changes in the levels of MERCs resident proteins; and third, modulation of signaling pathways in turn altering MERCs biology. Finally, we highlighted that some compounds already used in clinics exert their specific activity or side effects by impinging on MERCs biology. Whether this applies to other drugs as well is likely to be explored in the future, enhancing our knowledge about their tissue-specific action and offering the possibility to ameliorate their phenotypic outcomes.

## Supplementary Materials

The following are available online at https://www.mdpi.com/2073-4409/9/7/1637/s1, **Table S1**. In this table, we highlight the proteins that have been identified at MERCs by means of different proteomic studies. We highlight proteins that are either tissue-specific or common among mitochondria-associated membranes isolated from different cell types or organs.
